# Operational Strategies to Reduce Casualty Clinic Crowding: Findings From an Ophthalmology Audit and Re-Audit

**DOI:** 10.21315/mjms-03-2026-176

**Published:** 2026-06-30

**Authors:** Thiruvarasu Gunasekaran, Jeevindran Valaiyuthan, Iqbal Tajunisah

**Affiliations:** 1 Department of Ophthalmology, Faculty of Medicine, University of Malaya, 50603 Kuala Lumpur, Malaysia; 2 Department of Ophthalmology, Hospital Tengku Ampuan Afzan, Jalan Tanah Putih, 25100 Kuantan, Pahang, Malaysia

**Keywords:** ophthalmology casualty clinic, clinical audit, walk-in patients, patient flow management, appointment compliance, healthcare service efficiency

## Abstract

Efficient appointment management is essential for maintaining optimal patient flow in high-volume ophthalmology casualty services. A multitude of factors drive clinic overcrowding and delays in service delivery. This audit aimed to evaluate appointment management performance in an ophthalmology casualty clinic by analysing walk-in rates, overbooking practices, compliance with staggered appointment times, and clinic completion time. A prospective audit was conducted over 30 working days in the Ophthalmology Casualty Clinic at General Hospital Kuantan. Data were collected using a standardised daily form that captured walk-in attendance, scheduled appointment slots, patient arrival times, and the total time required for physicians to complete casualty consultations. Audit standards were defined as a walk-in rate of ≤ 20%, an overbooking rate of ≤ 10%, compliance with staggered appointment times of ≥ 80%, and clinic completion within 5 h. A total of 796 patients attended the clinic during the audit period. The walk-in rate was 43.2%, exceeding the predefined standard. Overbooking rate and compliance with staggered appointment times met the audit standards. The mean clinic completion time was 6.2 h, exceeding the target duration. Several improvement measures were implemented over a three-month period. These included introducing a triage checklist, allocating urgent follow-up slots, monitoring peak attendance trends, and increasing staffing during peak hours. A re-audit was subsequently conducted over a two-month period. The walk-in rate decreased to 25.4%, and the mean clinic completion time improved to 4.8 h. This audit demonstrates that targeted operational interventions may improve clinic efficiency and patient flow in ophthalmology casualty services.

## Introduction

Ophthalmology casualty clinics play an important role in providing urgent eye care for patients with acute ocular conditions. These services frequently manage a combination of scheduled follow-up patients and unscheduled walk-in cases. In high-volume government hospitals, efficient appointment scheduling, established operational policies (1), and patient flow management are essential to maintain quality of care (2) and reduce excessive waiting times.

Appointment systems (3) commonly utilise staggered time slots to distribute patients evenly throughout clinic hours. When patients adhere to their assigned time slots, clinic congestion may be minimised, and consultation efficiency may improve. However, operational challenges often arise due to walk-in attendance, appointment overbooking, and patient noncompliance with scheduled time slots. These factors (4) may disrupt clinic workflow and contribute to prolonged clinic operating hours.

Walk-in patients represent a particular challenge in casualty settings. Although some patients present with genuine ocular emergencies, others may attend casualty clinics for nonurgent conditions that could be managed through scheduled appointments. High walk-in volumes may therefore reduce the predictability of the daily clinic workload.

In addition to appointment compliance indicators, clinic completion time is an important operational parameter. Prolonged clinic hours may indicate inefficiencies in patient flow and increased workloads for healthcare personnel.

This audit was conducted to evaluate appointment management performance in the Ophthalmology Casualty Clinic at General Hospital Kuantan by assessing walk-in rate, overbooking practices, compliance with staggered appointment timing, and the time required for doctors to complete daily casualty consultations. A re-audit was subsequently performed following the implementation of improvement measures.

## Methods

This study was conducted as a prospective clinical audit in the Ophthalmology Casualty Clinic at General Hospital Kuantan.

### Initial Audit

Data were collected over 30 consecutive working days. All patients attending the casualty clinic during this period were included. A standardised daily audit form was used to record the following:

- Number of walk-in patients
- Number of patients with scheduled appointments
- Distribution of appointments across staggered hourly slots (8 to 9 AM, 9 to 10 AM, 10 to 11 AM, 11 AM to 12 PM, 12 to 1 PM)
- Actual patient arrival time
- Time at which the attending doctor completed the final patient consultation of the day

The following audit standards were defined:

- Walk-in rate ≤ 20%
- Overbooking rate ≤ 10%
- Compliance with staggered timing ≥ 80%
- Clinic completion time ≤ 5 h

The walk-in rate was calculated as the proportion of walk-in patients among the total daily attendance. The overbooking rate was defined as the proportion of appointments scheduled beyond the predetermined clinic capacity. Compliance with staggered timing was defined as arrival within ± 30 min of the assigned appointment time. Clinic completion time was measured as the interval from the clinic start time to completion of the final patient consultation.

### Re-Audit

A re-audit was over a two-month period beginning three months after the initial audit, following the implementation of improvement measures. These measures included the following:

- Development and implementation of a standardised triage checklist to differentiate true emergencies from nonurgent cases
- Allocation of dedicated daily slots for semi-urgent follow-up patients
- Monthly analysis of attendance trends by time slot
- Increase staffing in the triage area during peak walk-in periods

Data collection and analysis methods used in the re-audit were identical to those used in the initial audit.

## Results

### Initial Audit

During the 30-day audit period, 796 patients attended the ophthalmology casualty clinic. Of these, 344 patients presented as walk-ins, and 449 attended scheduled appointments. The walk-in rate was 43.2%, exceeding the predefined audit standard of ≤ 20%. The overbooking rate was 1.7%, which was within the acceptable standard of ≤ 10%. Compliance with staggered appointment timing was 83.5%, meeting the predefined standard of ≥ 80%. The mean clinic completion time was 6.2 h, exceeding the target duration of 5 h.

### Re-Audit

The re-audit conducted three months later included 1,912 patients over a two-month period. The walk-in rate decreased to 25.4% following the implementation of triage filtering and the allocation of urgent review slots. The overbooking rate was 1.9%, also remaining within the predefined standard. Compliance with staggered appointment timing was 84.1%, also remaining within the predefined standard. The mean clinic completion time improved to 4.8 h, meeting the target duration of ≤ 5 h. Figure 1 compares the operational parameters between the initial audit and the re-audit. As illustrated, a reduction in walk-in rates and clinic completion times was observed, while overbooking and staggered appointment compliance showed no significant change.

## Discussion

This clinical audit evaluated several operational parameters related to appointment management and patient flow in an ophthalmology casualty clinic. The initial audit identified a high walk-in rate that substantially exceeded the predefined standard. This finding reflects a common operational challenge in casualty clinics, where unscheduled patient attendance can significantly affect service delivery. Walk-in patients may present with a wide spectrum of ocular complaints, ranging from genuine emergencies to relatively minor conditions. When the proportion of walk-in patients is high, the daily clinic workload becomes less predictable. This unpredictability may result in overcrowding (4) and uneven distribution of patients throughout clinic hours, and may contribute to prolonged clinic operating times.[Table t1-14mjms3303_sc]

In the initial audit, the elevated walk-in rate was associated with a prolonged clinic completion time. Although overbooking was well controlled and compliance with staggered appointment timing (5) was acceptable, the large number of walk-in patients likely increased the total volume of patients requiring assessment by the attending physician.

After the implementation of targeted improvement measures, several operational changes were introduced. The triage checklist provided a structured approach for identifying patients requiring urgent ophthalmic evaluation. This approach may have reduced unnecessary casualty visits by redirecting nonurgent cases to scheduled clinic pathways. The allocation of dedicated urgent follow-up slots provided an alternative pathway for patients who required earlier review but did not require casualty evaluation. This strategy may have reduced reliance on walk-in visits.

In addition, monitoring attendance trends allowed the identification of peak walk-in periods that created workflow (6) bottlenecks. Increasing staffing in the triage area during these periods improved patient processing at the initial stage of clinic flow. These combined measures were associated with a reduction in the walk-in rate during the re-audit period. Importantly, the re-audit demonstrated that clinic completion time improved to within the predefined target despite a substantially higher patient volume. This finding suggests that improvements in patient flow processes may help mitigate the effects of increased clinic workload. Other operational indicators, such as the overbooking rate and compliance with staggered appointment timing, remained relatively stable during the re-audit period. This stability suggests that the scheduling system was already functioning adequately during the initial audit.

These findings highlight the importance of continuous monitoring of clinic operational parameters. Even when scheduling systems function appropriately, high levels of unscheduled patient attendance may significantly influence clinic efficiency. Implementing structured triage processes and flexible appointment pathways may therefore improve operations in high-volume ophthalmology casualty clinics.

## Conclusion

Crowding in casualty clinics is a common operational challenge in high-volume healthcare clinics. Effective crowd management is important for reducing waiting time and minimising burnout among healthcare personnel. Multiple operational parameters may influence clinic efficiency, including appointment scheduling, patient compliance with appointment times, overbooking practices, and the burden of walk-in patients.

In the initial audit, a high walk-in rate was identified as a major operational issue that contributed to prolonged clinic completion times. Following the implementation of targeted operational measures, a reduction in walk-in attendance and an improvement in clinic completion time were observed during the re-audit period despite a higher patient load.

These findings suggest that the walk-in burden is an important determinant of patient flow in ophthalmology casualty services. Continuous monitoring of operational indicators and periodic re-audits may help improve clinic efficiency and support sustainable crowd-management strategies.

## Figures and Tables

**Figure 1:**
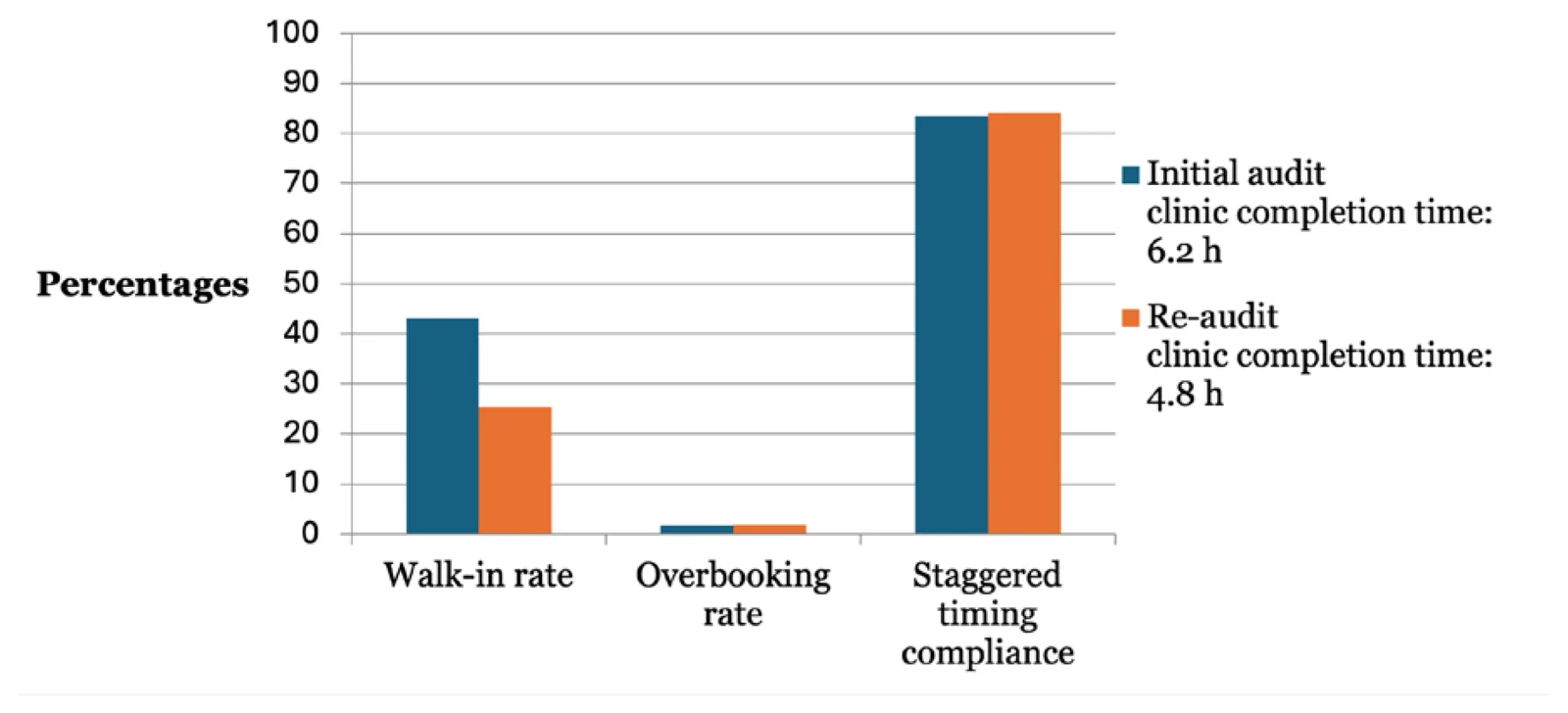
Comparison of operational parameters between the initial audit and re-audit.

**Table 1 t1-14mjms3303_sc:** Primary audit parameters and results during the 30-day audit period in the ophthalmology casualty clinic.

Parameter	Numerator (30 d)	Denominator (30 d)	Formula used	Result, %	Audit standard, %	Status
Walk-in rate	344 walk-in patients	796 total patients attended	(Walk-ins ÷ Total attended) × 100	43.2	≤ 20	Not achieved
Overbooking rate	10 excess bookings	600 total scheduled capacity	(Excess bookings ÷ Scheduled capacity) × 100	1.6	≤ 10	Achieved
Compliance to staggered timing	375 compliant arrivals	449 scheduled patients	(Compliant arrivals ÷ Scheduled patients) × 100	83.5	≥ 80	Achieved

## References

[1] Ministry of Health Malaysia (2024). Policies and procedures for outpatient services in Ministry of Health hospitals.

[2] World Health Organization (2006). Quality of care: a process for making strategic choices in health systems.

[3] Gupta D, Denton B (2008). Appointment scheduling in health care: challenges and opportunities. IIE Trans.

[4] Bahadori M, Teymourzadeh E, Ravangard R, Raadabadi M (2017). Factors affecting the overcrowding in outpatient healthcare. J Educ Health Promot.

[5] Neal RD, Hussain-Gambles M, Allgar VL, Lawlor DA, Dempsey O (2005). Reasons for and consequences of missed appointments in general practice in the UK: questionnaire survey and prospective review of medical records. BMC Fam Pract.

[6] De A, Gupta S, Chakraborty A (2024). Navigating patient flow: assessing the bottlenecks in out-patient services in a tertiary care hospital in India. Dr. Sulaiman Al Habib Med J.

